# Pharmacological inhibition of acetylcholinesterase improves the locomotion defective phenotype of a SCA3 *C. elegans* model

**DOI:** 10.17912/micropub.biology.001086

**Published:** 2024-02-06

**Authors:** Franziska Pohl, Victoria Lindsay-McGee, Paul Kong Thoo Lin, Patricia Maciel, Andreia Teixeira-Castro

**Affiliations:** 1 School of Pharmacy and Life Science, Robert Gordon University, Aberdeen, Scotland, United Kingdom; 2 Oncology/Dev. Biology, Washington University in St. Louis, St Louis, Missouri, United States; 3 The Royal (Dick) School of Veterinary Studies, University of Edinburgh, Edinburgh, Scotland, United Kingdom; 4 Life and Sciences Research Institute (ICVS), School of Medicine, University of Minho, Braga, Portugal; 5 Braga/Guimarães, Portugal, ICVS/3B’s-PT Government Associate Laboratory

## Abstract

Inhibition of acetylcholinesterase (AChE) is a common used treatment option for Alzheimer’s disease. However, there has been limited research on the potential use of AChE inhibitors for the treatment of Machado-Joseph disease (MJD)/Spinocerebellar Ataxia 3 (SCA3), in spite of the positive results using AChE inhibitors in patients with other inherited ataxias. MJD/SCA3, the most common form of dominant Spinocerebellar Ataxia worldwide, is caused by an expansion of the polyglutamine tract within the ataxin-3 protein, and is characterized by motor impairments. Our study shows that administration of the AChE inhibitor neostigmine is beneficial in treating the locomotion defective phenotype of a SCA3/MJD model of *C. elegans* and highlights the potential contribution of AChE enzymes to mutant ataxin-3-mediated toxicity.

**Figure 1. Pharmacological acetylcholinesterase inhibition ameliorates mutant ATXN3-medited motor defects in a SCA3 C. elegans model:**
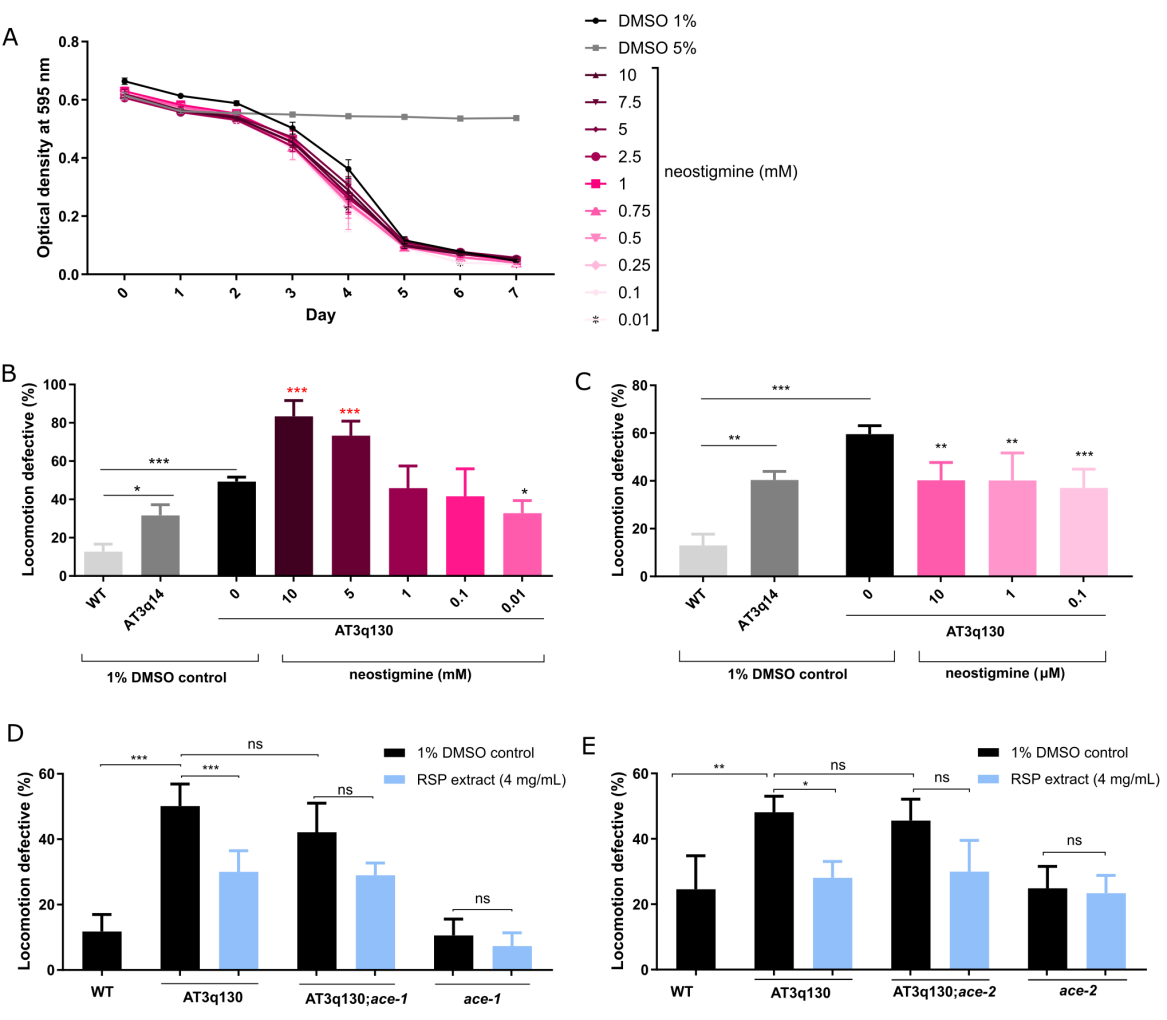
**A** : neostigmine (0.01-10.0 mM) shows no major toxic effects in *C. elegans* . Toxicity was assessed using the food clearance assay. The optical density of the OP50 suspension with neostigmne-treated animals ( N2 ) at the concentrations depicted, was measured daily. The mean OD was calculated for each day from five samples and plotted over time. Control DMSO (1%) corresponds to drug vehicle and DMSO at 5% was used as positive (toxic compound) control. **B** : Locomotion defective behaviour of AT3q130 animals, comparison between treated (neostigmine 0.01-10 mM) and untreated animals in comparison to wild type ( N2 ) and AT3q14 controls. Statistical significant difference determined using One-way ANOVA and Bonferroni’s multiple comparison analysis compared to AT3q130 control: ***p≤0.001 (decreased motility); *p≤0.05, ***p≤0.001; n=5 **C** : Locomotion defective behaviour of AT3q130 animals, comparison between treated (neostigmine 0.1-10 µM) and untreated animals in comparison to wild type ( N2 ) and AT3q14 controls. Statistical significant difference determined using One-way ANOVA and Bonferroni’s multiple comparison analysis compared to AT3q130 control: ***p≤0.001, **p≤0.01, n=5 **D** : Locomotion defective behaviour of AT3q130 animals compared to double mutant AT3q130; ace-1 and WT and ace-1 control. Statistical significant difference determined using One-way ANOVA and Bonferroni’s multiple comparison analysis: ***p≤0.001, ns-not significant; n=5 **E** : Locomotion defective behaviour of AT3q130 animals compared to double mutant AT3q130; ace-2 and WT and ace-2 control. Statistical significant difference determined using One-way ANOVA and Bonferroni’s multiple comparison analysis: *p≤0.05, **p≤0.01, ns-not significant; n=4

## Description

Inhibition of acetylcholinesterase (AChE) is currently one of the most used treatment options for Alzheimer’s disease (AD). The three main drugs galantamine, rivastigmine and donepezil are used to improve the choline deficiency found in AD patients (Mehta et al., 2012) . Also other diseases, such as for example myasthenia gravis are treated using AChE inhibitors i.e. neostigmine in combination with other treatment options (Gold et al., 2008) . Research on the potential use of AChE inhibitors for the treatment of Machado-Joseph disease (MJD)/Spinocerebelar Ataxia 3 (SCA3), an inherited neurodegenerative disorder characterized mostly by motor impairments, is limited. In a small (21 participants) double‐blind, triple‐crossover trial of oral physostigmine (AChE inhibitor) in inherited ataxias published by Kark et al. in 1981 (Kark et al., 1981) showed physostigmine to be more effective than the placebo control in treating the ataxia symptoms. More than half of the patients (13) showed statistically significant responses to physostigmine. The latter was a follow up on a study published in 1977 by the same lead author, where similar positive results were observed (Kark et al., 1977) . In contrast, in a later study (1997) using physostigmine, by Wessel et al *.* (Wessel et al., 1997) , no significant effect on cerebellar symptoms were detected when using a transdermal (patch) system. In both studies by Kark et al., oral administration or intravenous infusion was used (Kark et al., 1981, 1977) . The initial studies by Kark et al *.* (Kark et al., 1981, 1977) led us to believe that AChE inhibition should be further investigated as a potential treatment option for MJD/SCA3. This was further supported by our previous study, in which the AChE inhibition potential of both, an ethanolic Rape seed pomace (RSP) extract and its main secondary metabolite sinapine, was established (Yates et al., 2019) . Both, administration of the RSP extract and sinapine were able to improve the motility deficient phenotype of a *C. elegans* model of MJD/SCA 3 (Pohl et al., 2019) .

Acetylcholine (Ach) is the most widely used neurotransmitter in the *C. elegans* nervous system (Pereira et al., 2015) . Acetylcholine neurotransmission is ceased by enzymatic hydrolysis of Ach by acetylcholinesterase (AChE), in the synaptic cleft. *C. elegans* has four so far known ace genes encoding for AChE, *ace-1* to *-4* , compared to only one in vertebrates. These four genes encode three different pharmacological classes of AChE (A-C) (Combes et al., 2001, 2000) . The gene *ace-1* encodes AChE class A and produces most (~50%) of the AChE activity in the nematode (Johnson et al., 1981; Johnson and Russell, 1983) . *ace-1* mutants were found to be developmentally and behaviorally indistinguishable from wild type animals ( N2 ). The latter has 42% identity with human AChEs (Combes et al., 2000) . *ace-2* , is a structural gene for AChE class B (Culotti et al., 1981) . Mutations in *ace-2* seem to have no effect on the mobility of the animals. Mutants that harbor mutations in both *ace-1* and *ace-2* lack approximately 98% of their acetylcholinesterase activity and present an uncoordinated phenotype (Culotti et al., 1981) .

To determine whether AChE inhibition could be at least partially responsible for the improvement of the MJD disease phenotype in *C. elegans* (motility deficiency), in this study pharmacological and pharmacogenetic methods were employed.

For the pharmacological approach neostigmine was used, which was previously used as positive control in the *in vitro* analysis of AChE inhibition activity of the RSP extract (Yates et al., 2019) . The food clearance assay ( **Figure 1A** ) showed no toxicity of neostigmine towards *C. elegans* between 0.1 and 10 mM. Therefore, the same neostigmine concentrations were applied in the motility assay ( **Figure 1B** ).

Although 10 mM neostigmine did not seem to affect the worm’s development and fecundity as determined by the food clearance assay ( **Figure 1A** ), there was a significant increase in the percentage of mutant ataxin-3-expressing (AT3q130) animals with locomotion impairments when compared to the untreated control. The locomotion defect increased significantly from 49.32±2.32% (AT3q130, 1% DMSO) to 83.37±8.28% (10 mM neostigmine, **Figure 1B** ). A significantly worsening effect on the phenotype was also visible for 5 mM neostigmine (73.27±7.58%, **Figure 1B** ). Similar observations were made by Kalinnikova et al *.* (Kalinnikova et al., 2013) , where 6, 12, 24 mM neostigmine (90 minute exposure) showed increasing uncoordinated behavior while swimming in wild type *C. elegans* (after mechanical stimulus), due to aldicarb-like toxic effects. So even though concentrations up to 10 mM seem to be non-toxic ( **Figure 1A** ), they still reduce motility in WT as well as AT3q130 *C. elegans.*

Interestingly at lower neostigmine concentrations (0.1 and 1.0 mM) non-significant differences between treated and untreated (1% DMSO) AT3q130 animals were seen. At 0.01 mM a significant positive improvement of the locomotion behavior was observed. Due to the improving phenotype at the lowest concentration of neostigmine, further studies were initiated that included animals’ exposure to 10 µM (0.01 mM), 1 µM and 0.1 µM of the drug ( **Figure 1C** ). The results obtained for the lower neostigmine concentrations demonstrated significant motility improvement of the AT3q130 model for all 3 tested concentrations (0.1, 1.0 and 10.0 µM, **Figure 1C** ). To shed further light on the hypothesis that AChE inhibition of the RSP extract could be partially responsible for the improved motility phonotype, also a pharmacogenetic approach was employed.

For the pharmacogenetic approach, *ace-1* ( VC505 ; *ace-1* ( *ok663* ) X.) and *ace-2 (* RB1942 *; ace-2 ( ok2545 ) I.)* mutants were crossed with AT3q130 animals after initial backcrossing (6x) with WT animals. The double mutants, AT3q130; *ace-1* and AT3q130; *ace-2* were then tested in the motility assay together with the appropriate controls, i.e. WT, AT3q130 and *ace-1 / ace-2* mutant animals, untreated and treated with RSP extract ( **Figure 1D,E** ).

The *ace-1* mutant itself showed no significant changes in motility compared to the WT control in our assay ( **Figure 1D** ), although Melstrom and Williams (Melstrom and Williams, 2007) had previously suggested a decrease in the rate of movement for *ace-1* mutant animals. As previously shown in Pohl *at al.* (Pohl et al., 2019) , treatment with the RSP extract (4 mg/mL) significantly improved the motility deficient phenotype of AT3q130 animals and was used here as positive control ( **Figure 1D** ). The double mutant with approximately half the AChE activity showed no significant difference in motor performance when compared to the AT3q130, after 5 independent experiments. However, there is a visible trend towards a reduced motility impairment, with *e.g.* mean values of 50.12% and 42.11% for AT3q130 and AT3q130; *ace-1* respectively. This suggests a minor involvement of AChE activity in the motor behavior phenotype improvement shown by these animals. This was further improved upon treatment with RSP extract, resulting in a percentage of 28.96 % of locomotion defective animals, which was similar to the results obtained for the RSP extract treatment in the AT3q130 strain (30.05%, **Figure 1D** ).

The results obtained for the *ace-2* mutant ( **Figure 1E** ), show very similar results, i.e. *ace-2* itself does not seem to show a significant movement phenotype from WT. There is also no significant improvement of the AT3q130 movement phenotype in the *ace-2* mutant background, 48.15% vs. 45.58% respectively. The extract however improves the AT3q130 movement significantly from 48.15% to 28.08%. The extract also improves the motility in the AT3q130; *ace-2* double mutant from 45.58% to 29.95%, however, this improvement is not significant ( **Figure 1E** ). The extract has previously been shown to be specific to improve the motility of AT3q130 animals only and not that of WT or AT3q14 animals (Pohl et al., 2019) .

Overall, the pharmacogenetic results suggest that AChE inhibition contributes to the improvement of the SCA3/MJD-like motor phenotype in *C. elegans* , as seen by the partial amelioration of the animals’ motor phenotype in the background of *ace-1* loss of function. However, other *ace* enzymes may compensate for this activity, as this amelioration does not reach statistical significance. Moreover, the RSP extract has previously been shown to act through the activation of GST-4 , a detoxification enzyme downstream of *skn-1* (Franziska Pohl et al., 2019), this might explain the observed further improvement of the double mutant (AT3q130; *ace-1* ). However, the fact that in both cases a slight trend towards motility improvement was observed led to the conclusion that AChE might play a role in the effect observed by the RSP extracts but further investigations will be required. This is the first study looking into the importance of AChE inhibition *via* drug treatment and decreased expression of AChE *via* gene knockout in the context of a MJD/SCA3 *C. elegans* model.

To further investigate our hypothesis, neostigmine should be tested in WT as well as in AT3q14 animals to show whether it is specific to AT3q130 animals as previously shown for the RSP (Pohl et al., 2019) . It would also be useful to determine if the RSP extractis causing a significant decrease of AChE activity *in vivo* within the SCA3/MJD model as well as in wild type ( N2 ) animals. In addition, it would be interesting to create triple mutants of both AT3q130; *ace-1* and AT3q130; *ace-2* animals with *ace-3* . The addition of knocking out an extra gene encoding approximately 5% more AChE activity (Combes et al., 2000) , in *C. elegans* , might lead to additional improvements. Similarly, the treatment of the double mutants AT3q130; *ace-1* and AT3q130; *ace-2* with neostigmine might be beneficial. Data from a triple mutant including AT3q130, *ace-1* and *ace-2* could be difficult to interpret, due to the fact that the double mutant ( *ace-1* ; *ace-2* ) are relatively uncoordinated (Culotti et al., 1981) , which would interfere with the already motility impaired AT3q130 model, allowing only to infer additional activities of the RSP extract.

## Methods

*Strains and general maintenance*

All strains ( **Table 1** ) were cultured and observed using standard methods (Brenner, 1974) unless otherwise stated. *C. elegans* grew on nematode growth medium (NGM) plates seeded with *Escherichia coli* OP50 strain at 20°C. All the strains were backcrossed to Bristol strain N2 six to eight times. The MJD related strains AT3q14 (AM510 ( *rmIs228* [P *rgef-1* ::AT3v1-1q14::yfp])) , and AT3q130 (AM685 ( *rmIs263* [P *rgef-1* ::AT3v1-1q130::yfp] II)) were previously described (Teixeira-Castro et al., 2011) and double mutant strains (AT3q130; *ace-1* and AT3q130; *ace-2* ) were generated using common breeding techniques (Fay, 2013) . The remaining strains were provided by the *Caenorhabditis Genetics Center* (CGC).

**Table 1 *C. elegans* strains used in this study**

**Table:** 

**STRAIN NAME**	**NAME IN PAPER**	**GENOTYPE**	**SOURCE**	**NOTES/REFERENCE**
**N2**	N2 /WT	wt ( *C. elegans* wild isolate)	CGC	Brenner, 1974
**AM510**	AT3q14	*rmIs228* [P *rgef-1* ::AT3v1-1q14::yfp]	Morimoto lab	Teixeira-Castro et al., 2011
**AM685**	AT3q130	*rmIs263* [P *rgef-1* ::AT3v1-1q130::yfp] II	Morimoto lab	Teixeira-Castro et al., 2011
**MAC054**	*ace-1*	*ace-1 ( ok663 )* X	Maciel lab	this study, 6x back crossed VC505 ( *ok663* )
**MAC090**	*ace-2*	*ace-2 ( ok2545 )* I	Maciel lab	this study, 6x back crossed RB1942 ( *ok2545* )
**MAC217**	AT3q130; *ace-1*	*rmIs263* [P *rgef-1* ::AT3v1-1q130::yfp] II; *ace-1 ( ok663 )* X	Maciel lab	this study, cross between AM685 and MAC054
**MAC055**	*ace-2 ;* AT3q130	*ace-2 ( ok2545 )* I; *rmIs263* [P *rgef-1* ::AT3v1-1q130::yfp] II	Maciel lab	this study, cross between AM685 and MAC090

*Rapeseed pomace extract*

The RSP extract was prepared as previously described (Pohl et al., 2018; Yates et al., 2019) . Several extractions were performed, and the obtained extracts collected, combined, homogenized, vacuum packed and stored at -80°C. The extract was the same as described in Pohl et al *.* (Pohl et al., 2019) .

*C. elegans drug toxicity assay*

The toxicity of distinct concentrations of neostigmine *in vivo* was determined in the wild-type N2 Bristol strain, using the food clearance assay (Voisine et al., 2007) . The assay was performed as previously described (Teixeira-Castro et al., 2015; Voisine et al., 2007) in liquid culture in 96-well plate format using concentrations from 0.01-10 mM of neostigmine, using DMSO as the drug vehicle at a final concentration of 1%. Animals treated with 1% and 5% DMSO were used as a non-toxic (vehicle control) and as a toxic concentration control, respectively.

*Motor performance of the C. elegans MJD model treated with RSP extract*

AT3q130 animals were treated with concentrations of neostigmine ranging from 10.0 mM to 0.1 μM in liquid culture in 96-well format as described for the toxicity assay (Voisine et al., 2007) . The motility assay was performed as previously described (Teixeira-Castro et al., 2011) using *C. elegans* strains expressing WT (AT3q14) and mutant ATXN3 (AT3q130) proteins in their nervous system, as well as N2 as WT control. In the pharmacogenetic assays, *ace-1* and *ace-2* mutants were used as additional controls. The effect of the RSP extract in AT3q130 animals was tested in the presence or absence of these enzymes, as previously described (Teixeira-Castro et al., 2015) .

*Statistical analysis*

All statistical analyses were performed using GraphPad Prism 7 (Version 7.01). Continuous variables were tested for normal distribution (Shapiro-Wilk or Kolmogorov-Smirnov normality test) and outliers; and were then analyzed with two-way ANOVA, using Bonferroni’s multiple comparison analysis for post hoc comparison. A critical value for significance of p≤0.05 was applied throughout the study. All experiments were run in quadruplicate or quintuplicate (n=4 or 5) and data presented are showing mean ± standard deviation.
